# Dichlorido{*N*,*N*-dimethyl-*N*′-[1-(2-pyrid­yl)ethyl­idene]ethane-1,2-diamine-κ^3^ *N*,*N*′,*N*′′}copper(II)

**DOI:** 10.1107/S1600536810011712

**Published:** 2010-04-10

**Authors:** Muhammad Saleh Salga, Hamid Khaledi, Hapipah Mohd Ali, Rustam Puteh

**Affiliations:** a Department of Chemistry, University of Malaya, 50603 Kuala Lumpur, Malaysia; b Department of Physics, University of Malaya, 50603 Kuala Lumpur, Malaysia

## Abstract

In the title compound, [CuCl_2_(C_11_H_17_N_3_)], the Cu^II^ ion is five-coordinated with a distorted square-pyramidal configuration. The three N atoms of the Schiff base ligand and one Cl atom are located in the basal plane, whereas the other Cl atom is apically positioned.

## Related literature

For the crystal structures of similar copper (II) complexes, see: Wang *et al.* (2009 ▶); Yuan & Zhang (2005 ▶); Zhang *et al.* (2009 ▶). For a description of the geometry of five-coordinated metal complexes, see: Addison *et al.* (1984 ▶).
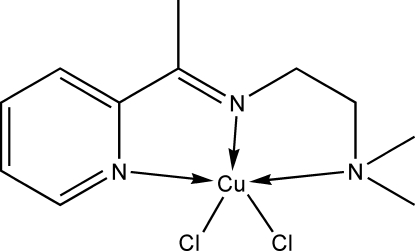


## Experimental

### 

#### Crystal data

[CuCl_2_(C_11_H_17_N_3_)]
*M* *_r_* = 325.72
Orthorhombic, 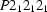

*a* = 9.81448 (12) Å
*b* = 9.90297 (13) Å
*c* = 14.21414 (18) Å
*V* = 1381.51 (2) Å^3^
*Z* = 4
Mo *K*α radiation
μ = 1.95 mm^−1^
*T* = 100 K
0.30 × 0.23 × 0.07 mm

#### Data collection

Bruker APEXII CCD diffractometer
Absorption correction: multi-scan (*SADABS*; Sheldrick, 1996 ▶) *T*_min_ = 0.592, *T*_max_ = 0.876
10998 measured reflections
2439 independent reflections
2378 reflections with *I* > 2σ(*I*)
*R*_int_ = 0.026

#### Refinement

*R*[*F* ^2^ > 2σ(*F* ^2^)] = 0.017
*wR*(*F* ^2^) = 0.043
*S* = 1.03
2439 reflections
157 parameters
H-atom parameters constrained
Δρ_max_ = 0.21 e Å^−3^
Δρ_min_ = −0.20 e Å^−3^
Absolute structure: Flack (1983 ▶), 1022 Friedel pairs
Flack parameter: 0.010 (9)

### 

Data collection: *APEX2* (Bruker, 2007 ▶); cell refinement: *SAINT* (Bruker, 2007 ▶); data reduction: *SAINT*; program(s) used to solve structure: *SHELXS97* (Sheldrick, 2008 ▶); program(s) used to refine structure: *SHELXL97* (Sheldrick, 2008 ▶); molecular graphics: *X-SEED* (Barbour, 2001 ▶); software used to prepare material for publication: *publCIF* (Westrip, 2010 ▶).

## Supplementary Material

Crystal structure: contains datablocks I, global. DOI: 10.1107/S1600536810011712/pv2266sup1.cif

Structure factors: contains datablocks I. DOI: 10.1107/S1600536810011712/pv2266Isup2.hkl

Additional supplementary materials:  crystallographic information; 3D view; checkCIF report

## Figures and Tables

**Fig. 1.**
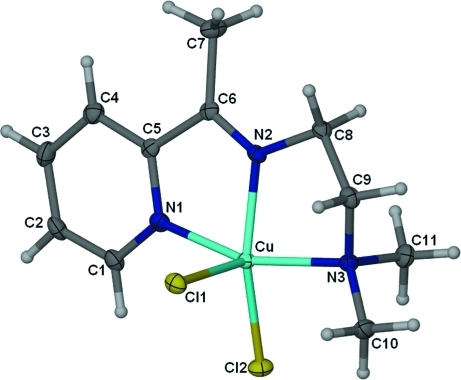
Thermal ellipsoid plot of the title compound at the 50% probability level.

## supplementary crystallographic information

### Comment

The title compound was obtained by the reaction of *N,N*-dimethyl-*N'*-[methyl(2-pyridyl)methylene]ethane-1,2-diamine with copper(II) chloride. In the molecule of the complex, the metal ion is penta-coordinated by the tridentate Schiff base ligand and two chloride atoms (Fig. 1). The geometry of the complex can be determined by using the index τ = (β-α)/60, where β is the largest angle and α is the second one around the metal center. For an ideal square-pyramidal geometry τ is 0, while it is 1 in a perfect trigonal-bipyramid (Addison *et al.*,1984). The two largest angels in the title compound are 158.45 (6)° (N1—Cu—N3) and 154.98 (5)° (N2—Cu—Cl2) which give a τ value of 0.058. This value indicates a slightly distorted square pyramidal geometry in which the three N atoms of the Schiff base ligand and one chloride atom occupy the basal positions and the other chloride atom is placed in the apical position.

### Experimental

The Schiff base ligand was prepared *via *condensation reaction of *N*,*N*-dimethylethyldiamine (0.44 g, 5 mmol) and 2-acetylpyridine (0.61 g, 5 mmol) by refluxing in ethanol (50 ml) for 2 h. For synthesis of the title complex a mixture of the Schiff base ligand (0.57 g, 3 mmol) and copper (II) chloride dihydrate (0.51 g, 3 mmol) in ethanol (50 ml) was stirred at room temperature for half an hour. The solvent was then evaporated partially to yield the title complex as a green solid. Suitable crystals for X-ray crystallography were obtained upon slow evaporation of an ethanolic solution at room temperature.

### Refinement

Hydrogen atoms were placed at calculated positions (C—H 0.95-0.98 Å), and were treated as riding on their parent atoms, with U_iso_(H) set to 1.2-1.5 times U_eq_(C). An absolute structure was established using anomalous dispersion effects; 1021 Friedel pairs were not merged.

### Crystal data

**Table:**

[CuCl_2_(C_11_H_17_N_3_)]	*F*(000) = 668
*M**_r_* = 325.72	*D*_x_ = 1.566 Mg m^−^^3^
Orthorhombic, *P*2_1_2_1_2_1_	Mo *K*α radiation, λ = 0.71073 Å
Hall symbol: P 2ac 2ab	Cell parameters from 7155 reflections
*a* = 9.81448 (12) Å	θ = 2.5–30.4°
*b* = 9.90297 (13) Å	µ = 1.95 mm^−^^1^
*c* = 14.21414 (18) Å	*T* = 100 K
*V* = 1381.51 (2) Å^3^	Block, green
*Z* = 4	0.30 × 0.23 × 0.07 mm

### Data collection

**Table:**

Bruker APEXII CCD diffractometer	2439 independent reflections
Radiation source: fine-focus sealed tube	2378 reflections with *I* > 2σ(*I*)
graphite	*R*_int_ = 0.026
φ and ω scans	θ_max_ = 25.0°, θ_min_ = 2.5°
Absorption correction: multi-scan (*SADABS*; Sheldrick, 1996)	*h* = −11→11
*T*_min_ = 0.592, *T*_max_ = 0.876	*k* = −11→11
10998 measured reflections	*l* = −16→16

### Refinement

**Table:**

Refinement on *F*^2^	Secondary atom site location: difference Fourier map
Least-squares matrix: full	Hydrogen site location: inferred from neighbouring sites
*R*[*F*^2^ > 2σ(*F*^2^)] = 0.017	H-atom parameters constrained
*wR*(*F*^2^) = 0.043	*w* = 1/[σ^2^(*F*_o_^2^) + (0.0245*P*)^2^] where *P* = (*F*_o_^2^ + 2*F*_c_^2^)/3
*S* = 1.03	(Δ/σ)_max_ = 0.001
2439 reflections	Δρ_max_ = 0.21 e Å^−^^3^
157 parameters	Δρ_min_ = −0.20 e Å^−^^3^
0 restraints	Absolute structure: Flack (1983), 1022 Friedel pairs
Primary atom site location: structure-invariant direct methods	Flack parameter: 0.010 (9)

### Special details

**Table:**

Geometry. All esds (except the esd in the dihedral angle between two l.s. planes) are estimated using the full covariance matrix. The cell esds are taken into account individually in the estimation of esds in distances, angles and torsion angles; correlations between esds in cell parameters are only used when they are defined by crystal symmetry. An approximate (isotropic) treatment of cell esds is used for estimating esds involving l.s. planes.
Refinement. Refinement of *F*^2^ against ALL reflections. The weighted *R*-factor wR and goodness of fit *S* are based on *F*^2^, conventional *R*-factors *R* are based on *F*, with *F* set to zero for negative *F*^2^. The threshold expression of *F*^2^ > σ(*F*^2^) is used only for calculating *R*-factors(gt) etc. and is not relevant to the choice of reflections for refinement. *R*-factors based on *F*^2^ are statistically about twice as large as those based on *F*, and *R*- factors based on ALL data will be even larger.

### Fractional atomic coordinates and isotropic or equivalent isotropic displacement parameters (Å^2^)

**Table:**

	*x*	*y*	*z*	*U*_iso_*/*U*_eq_	
Cu	0.80028 (2)	0.16329 (2)	0.099598 (15)	0.01175 (7)	
Cl1	0.56577 (5)	0.08033 (5)	0.13120 (3)	0.01619 (11)	
Cl2	0.92633 (5)	0.14346 (5)	0.23208 (3)	0.01790 (12)	
N1	0.86920 (16)	−0.00757 (17)	0.03434 (12)	0.0139 (4)	
N2	0.76828 (16)	0.20963 (16)	−0.03353 (11)	0.0124 (4)	
N3	0.75938 (15)	0.36500 (16)	0.11931 (11)	0.0132 (4)	
C1	0.9300 (2)	−0.11396 (19)	0.07379 (15)	0.0169 (4)	
H1	0.9448	−0.1136	0.1398	0.020*	
C2	0.9722 (2)	−0.2245 (2)	0.02225 (15)	0.0185 (5)	
H2	1.0174	−0.2976	0.0522	0.022*	
C3	0.9479 (2)	−0.2274 (2)	−0.07327 (15)	0.0200 (5)	
H3	0.9737	−0.3036	−0.1098	0.024*	
C4	0.8846 (2)	−0.1161 (2)	−0.11553 (15)	0.0175 (4)	
H4	0.8666	−0.1155	−0.1812	0.021*	
C5	0.84897 (19)	−0.0075 (2)	−0.06026 (13)	0.0131 (4)	
C6	0.79321 (18)	0.12257 (18)	−0.09696 (13)	0.0129 (4)	
C7	0.7771 (2)	0.1485 (2)	−0.20048 (13)	0.0200 (5)	
H7A	0.6843	0.1805	−0.2132	0.030*	
H7B	0.7935	0.0648	−0.2354	0.030*	
H7C	0.8428	0.2173	−0.2204	0.030*	
C8	0.71958 (19)	0.3470 (2)	−0.05216 (13)	0.0139 (4)	
H8A	0.6464	0.3460	−0.1001	0.017*	
H8B	0.7949	0.4047	−0.0751	0.017*	
C9	0.6657 (2)	0.3992 (2)	0.04119 (14)	0.0158 (4)	
H9A	0.6548	0.4985	0.0377	0.019*	
H9B	0.5751	0.3592	0.0536	0.019*	
C10	0.6923 (2)	0.3973 (2)	0.20995 (13)	0.0195 (4)	
H10A	0.7533	0.3737	0.2620	0.029*	
H10B	0.6076	0.3456	0.2154	0.029*	
H10C	0.6718	0.4941	0.2124	0.029*	
C11	0.8874 (2)	0.4429 (2)	0.11176 (15)	0.0200 (5)	
H11A	0.9314	0.4230	0.0514	0.030*	
H11B	0.9486	0.4176	0.1633	0.030*	
H11C	0.8671	0.5396	0.1155	0.030*	

### Atomic displacement parameters (Å^2^)

**Table:**

	*U*^11^	*U*^22^	*U*^33^	*U*^12^	*U*^13^	*U*^23^
Cu	0.01269 (12)	0.01237 (12)	0.01020 (12)	0.00077 (10)	−0.00026 (10)	0.00045 (10)
Cl1	0.0134 (2)	0.0185 (3)	0.0166 (2)	−0.0024 (2)	−0.00053 (18)	0.0027 (2)
Cl2	0.0177 (2)	0.0229 (3)	0.0130 (2)	0.0021 (2)	−0.00368 (19)	0.0008 (2)
N1	0.0115 (8)	0.0159 (9)	0.0143 (8)	−0.0012 (7)	0.0026 (7)	0.0014 (7)
N2	0.0107 (9)	0.0133 (8)	0.0131 (8)	0.0003 (6)	−0.0012 (7)	0.0033 (7)
N3	0.0137 (8)	0.0146 (8)	0.0114 (8)	0.0001 (6)	0.0009 (6)	0.0000 (7)
C1	0.0181 (10)	0.0146 (10)	0.0180 (11)	−0.0018 (8)	0.0028 (9)	0.0036 (8)
C2	0.0203 (11)	0.0136 (11)	0.0217 (12)	0.0004 (8)	0.0034 (9)	0.0057 (9)
C3	0.0229 (11)	0.0144 (10)	0.0227 (11)	−0.0009 (9)	0.0024 (9)	−0.0051 (9)
C4	0.0170 (10)	0.0184 (11)	0.0171 (11)	−0.0034 (8)	−0.0009 (9)	−0.0014 (9)
C5	0.0101 (10)	0.0148 (10)	0.0143 (10)	−0.0034 (8)	0.0011 (8)	−0.0012 (8)
C6	0.0093 (9)	0.0160 (9)	0.0134 (9)	−0.0037 (7)	−0.0012 (9)	−0.0009 (8)
C7	0.0223 (11)	0.0237 (11)	0.0139 (10)	0.0061 (10)	−0.0012 (8)	−0.0014 (9)
C8	0.0145 (10)	0.0136 (10)	0.0137 (10)	0.0008 (9)	−0.0019 (7)	0.0020 (8)
C9	0.0146 (10)	0.0144 (10)	0.0185 (10)	0.0017 (8)	−0.0009 (8)	0.0019 (9)
C10	0.0272 (11)	0.0176 (10)	0.0136 (10)	0.0018 (10)	0.0029 (10)	−0.0021 (8)
C11	0.0213 (11)	0.0183 (11)	0.0204 (12)	−0.0064 (8)	−0.0018 (9)	−0.0015 (10)

### Geometric parameters (Å, °)

**Table:**

Cu—N2	1.9723 (16)	C4—C5	1.377 (3)
Cu—N1	2.0447 (17)	C4—H4	0.9500
Cu—N3	2.0567 (16)	C5—C6	1.494 (3)
Cu—Cl2	2.2617 (5)	C6—C7	1.502 (3)
Cu—Cl1	2.4848 (5)	C7—H7A	0.9800
N1—C1	1.334 (3)	C7—H7B	0.9800
N1—C5	1.359 (2)	C7—H7C	0.9800
N2—C6	1.271 (2)	C8—C9	1.519 (3)
N2—C8	1.466 (3)	C8—H8A	0.9900
N3—C11	1.478 (2)	C8—H8B	0.9900
N3—C10	1.482 (2)	C9—H9A	0.9900
N3—C9	1.481 (2)	C9—H9B	0.9900
C1—C2	1.381 (3)	C10—H10A	0.9800
C1—H1	0.9500	C10—H10B	0.9800
C2—C3	1.379 (3)	C10—H10C	0.9800
C2—H2	0.9500	C11—H11A	0.9800
C3—C4	1.401 (3)	C11—H11B	0.9800
C3—H3	0.9500	C11—H11C	0.9800
			
N2—Cu—N1	79.04 (7)	N1—C5—C6	113.54 (17)
N2—Cu—N3	82.74 (6)	C4—C5—C6	124.56 (17)
N1—Cu—N3	158.45 (6)	N2—C6—C5	114.06 (16)
N2—Cu—Cl2	154.98 (5)	N2—C6—C7	123.94 (18)
N1—Cu—Cl2	97.17 (5)	C5—C6—C7	121.90 (16)
N3—Cu—Cl2	94.45 (4)	C6—C7—H7A	109.5
N2—Cu—Cl1	95.91 (5)	C6—C7—H7B	109.5
N1—Cu—Cl1	96.59 (5)	H7A—C7—H7B	109.5
N3—Cu—Cl1	96.65 (4)	C6—C7—H7C	109.5
Cl2—Cu—Cl1	109.113 (18)	H7A—C7—H7C	109.5
C1—N1—C5	118.77 (18)	H7B—C7—H7C	109.5
C1—N1—Cu	127.64 (14)	N2—C8—C9	105.76 (15)
C5—N1—Cu	113.59 (13)	N2—C8—H8A	110.6
C6—N2—C8	124.33 (16)	C9—C8—H8A	110.6
C6—N2—Cu	119.47 (13)	N2—C8—H8B	110.6
C8—N2—Cu	116.17 (12)	C9—C8—H8B	110.6
C11—N3—C10	109.14 (15)	H8A—C8—H8B	108.7
C11—N3—C9	110.72 (15)	N3—C9—C8	111.17 (16)
C10—N3—C9	109.07 (15)	N3—C9—H9A	109.4
C11—N3—Cu	109.33 (12)	C8—C9—H9A	109.4
C10—N3—Cu	114.53 (12)	N3—C9—H9B	109.4
C9—N3—Cu	103.96 (12)	C8—C9—H9B	109.4
N1—C1—C2	122.50 (19)	H9A—C9—H9B	108.0
N1—C1—H1	118.8	N3—C10—H10A	109.5
C2—C1—H1	118.8	N3—C10—H10B	109.5
C3—C2—C1	119.1 (2)	H10A—C10—H10B	109.5
C3—C2—H2	120.4	N3—C10—H10C	109.5
C1—C2—H2	120.4	H10A—C10—H10C	109.5
C2—C3—C4	118.9 (2)	H10B—C10—H10C	109.5
C2—C3—H3	120.6	N3—C11—H11A	109.5
C4—C3—H3	120.6	N3—C11—H11B	109.5
C5—C4—C3	118.86 (19)	H11A—C11—H11B	109.5
C5—C4—H4	120.6	N3—C11—H11C	109.5
C3—C4—H4	120.6	H11A—C11—H11C	109.5
N1—C5—C4	121.81 (19)	H11B—C11—H11C	109.5
